# Software Defined Doppler Radar as a Contactless Multipurpose Microwave Sensor for Vibrations Monitoring

**DOI:** 10.3390/s17010115

**Published:** 2017-01-08

**Authors:** Antonio Raffo, Sandra Costanzo, Giuseppe Di Massa

**Affiliations:** Department of Computer Engineering, Modelling, Electronics, and Systems Science (DIMES), University of Calabria, 87036 Rende, Italy; a.raffo@dimes.unical.it (A.R.); giuseppe.dimassa@unical.it (G.D.M.)

**Keywords:** Doppler radar, SDR, vibration monitoring

## Abstract

A vibration sensor based on the use of a Software-Defined Radio (SDR) platform is adopted in this work to provide a contactless and multipurpose solution for low-cost real-time vibrations monitoring. In order to test the vibration detection ability of the proposed non-contact method, a 1 GHz Doppler radar sensor is simulated and successfully assessed on targets at various distances, with various oscillation frequencies and amplitudes. Furthermore, an SDR Doppler platform is practically realized, and preliminary experimental validations on a device able to produce a harmonic motion are illustrated to prove the effectiveness of the proposed approach.

## 1. Introduction

Techniques for detection of vibrations claim a wide range of applications, especially in the industrial and civil contexts, but also in biomedical engineering and security. There are different methods to analyze this kind of phenomenon, each basically suitable for monitoring a certain type of event. In the industrial field, the accurate vibration control of rotating machinery plays an important role in the prevention of failures of production plants. In this context, the most commonly used technologies adopt microelectromechanical systems (MEMS) or piezoelectric sensors, which are placed in direct contact with the activity source to be monitored. The mentioned approaches allow for achieving good results [1,2,3], but being directly subject to mechanical stresses, the sensors are exposed to wear and thus to a progressive alteration during their operation time. Optical methods, implemented with optical fibers placed at small distance from the monitored items, allow for obtaining higher reliability [4,5,6,7], but similarly to the piezoelectric sensors, they generally offer operating bandwidths on the order of a few kHz [8,9], and require the use of sophisticated signal analyzers to obtain good resolutions in results.

A different solution to the use of punctual position sensors, in the context of vibrations monitoring, is given by the use of remote sensors based on coherent radars. The use of Doppler radar techniques is increasingly popular for monitoring vibrations in the civil context, such as in the remote monitoring of the dynamic characteristics of buildings [10,11]. Similar applications can be found in the biomedical field for the development of physiological sensors able to monitor breathing and heart rate [12,13]. However, the implementation of a Doppler radar includes the use of laboratory test equipment or custom hardware on printed/integrated circuits, and this makes the system rather bulky and expensive [14]. Even if a lot of contributions exist in literature, all of them are based on standard hardware architectures, and are thus not able to change the operating frequency band and the related parameters in real time. In Reference [15], a classical continuous-wave (CW) Doppler radar configuration is assumed, and the problem of amplitude and distance dependency of noncontact vibration measurements is faced. Then, an extended differentiate and cross-multiply (DACM) algorithm is proposed and investigated, adopting the arctangent demodulation approach to recover the phase shift caused by the movement of the object. As it will be discussed in the next section, we completely avoid the codomain restriction due to the standard arctangent method by adopting the fast Fourier transform (FFT) to retrieve the phase shift related to the target vibration instead. In Reference [16], the modulation sensitivity of Doppler radar, again assumed in the classical hardware configuration, is investigated, by discussing the opportunity to adopt high microwave bands, which, however, result in being bulky in size and power consuming. To perform the sensitivity tests at two different frequencies, two distinct hardware radar sensors are realized in [16], thus increasing the overall cost. In Reference [17], a CW hardware configuration including three antennas and four receiver modules is realized and tested for tracking multiple humans in three-dimensional space, namely azimuth, elevation and range. Once again, the operating frequency is fixed once, limited by the hardware components. In this work, a completely new approach for Doppler radar implementation, fully based on a software-defined platform, is proposed. This alternative, flexible and low cost solution can be obtained through the use of a Software-Defined Radio (SDR) transceiver [18,19], which leads to implementing a multi-function radar, known as a Software Defined Radar (SDRadar) [20,21], composed of Radio Frequency (RF) hardware modules fully reconfigurable via software. An SDRadar system allows for realizing most of the basic operations (e.g., modulation, demodulation, filtering and mixing) by the simple use of programmable software modules, instead of specific hardware components [19], thus leading to a faster and cheaper development and manufacturing, as compared to conventional custom radars [22]. The choice of a software instead of a hardware platform is performed in this work to overcome the limits imposed by electronic circuitries. As a matter of fact, while architectonic structures limit the performances in terms of detectable frequency, due to the specific (fixed) adopted hardware, our solution is highly flexible. In particular, SDRadar is fully able to satisfy the frequency detection requirements, even in the presence of very low values (e.g., typical of heart oscillations), by simply changing via software the bandwidth, and thus the frequency detection range and the resolution. Even if many Printed Circuit Board (PCB) and chip level realizations of custom radar sensors can be actually found at a low price, the main benefit derived from our SDRadar solution is the demonstration of using programmable non-custom-designed RF equipment for radar motion detection study. This will enable researchers without radio frequency/microwave circuit backgrounds to study the signal processing, system consideration, and potential applications for microwave motion sensors. The high flexibility of the proposed software architecture, essentially related to the possibility of carrying out fast detections without the need to use wearable sensors or instruments in direct contact with the item to be monitored, makes these types of systems suitable to the detection of vibrations originating from different phenomena, as those generated by industrial plants. Moreover, the proposed contactless approach can be successfully adopted for the monitoring of vital parameters, with application in security systems based on the body motion detection, or in those emergency situations for the detection of people buried under critical conditions.

## 2. Continuous Wave Doppler Radar System: Detection Principle

**Figure 1:**
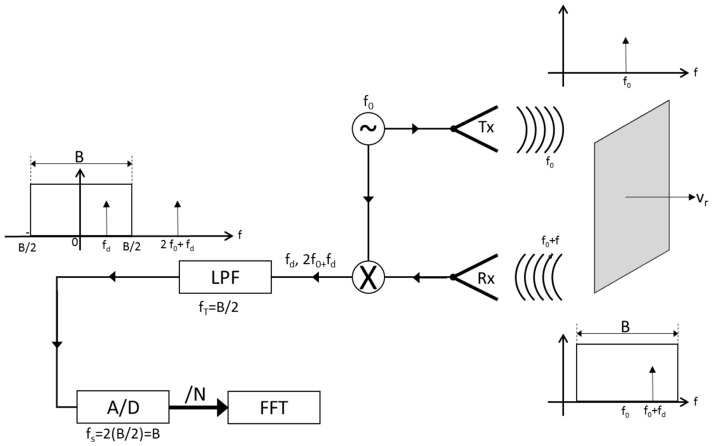
Continuous Wave (CW) Doppler radar architecture.

Any wave reflected from a moving object is subject to a frequency shift proportional to the object speed, due to a Doppler effect. This phenomenon is exploited by Doppler radars to detect moving targets. Let us suppose having a CW radar system that transmits a single tone signal (Equation (1)), at frequency *f*_0_, which has an impact against a target at nominal distance *d*_0_, causing a backscattering. The signal received by the radar is correlated to the transmitted one by a time delay τ (Equation (2)). This is, in turn, related to the nominal distance of the target, and also influenced by a frequency shift *f_d_* proportional to the radial velocity *v_r_* (Equation (3)) of the intercepted object. The information on the target speed can be extracted by performing the product of the received (Equation (4)) and the transmitted signals. When these latter are mixed and then filtered, a resulting baseband signal with a frequency linearly related to the radial velocity of the target (Figure 1) is obtained.

(1) $$t_{x}\left(t\right)=sin\left(2\pi f_{0}t\right),$$

(2) $$\tau \left(t\right)=\frac{2d_{0}}{c}+\frac{2v_{r}}{c}t,$$

(3) $$f_{d}=-\frac{2v_{r}}{\lambda },$$

(4) $$r_{x}\left(t\right)=A_{r}t_{x}\left(t-\tau \right)=A_{r}sin\left[2\pi f_{0}\left(t-\tau \left(t\right)\right)\right]=A_{r}\sin\left[2\pi f_{0}t-2\pi f_{0}\tau \left(t\right)\right]\;\;=A_{r}sin\left\{\left[2\pi \left(f_{0}+f_{d}\right)t\right]-\frac{4\pi d_{0}}{\lambda }\right\}.$$

After performing the Analog-to-Digital (A/D) conversion of the filtered signal, the frequency shift *f_d_*, detected by an FFT algorithm is obtained. Finally, Equation (3) can be applied to retrieve the radial velocity of the intercepted object.

The CW Doppler radar described above (Figure 1) is characterized by several important parameters, namely:

- *B*, which is the bandwidth of the receiving antenna;
- *f_T_*, giving the cutoff frequency of the low-pass filter, and constrained to the processing capacity of the system by the relationship:
  (5) $$f_{T}\geq \frac{B}{2},$$
- *f_S_*, giving the sampling frequency of the A/D circuit, and chosen to satisfy the Nyquist–Shannon sampling theorem:
  (6) $$f_{S}=2f_{T}\geq 2\frac{B}{2}\geq B,$$
- *N*, which is the number of samples collected and transmitted to the FFT processing.

## 3. Continuous Wave Doppler Radar for Vibration Detection

**Figure 2:**
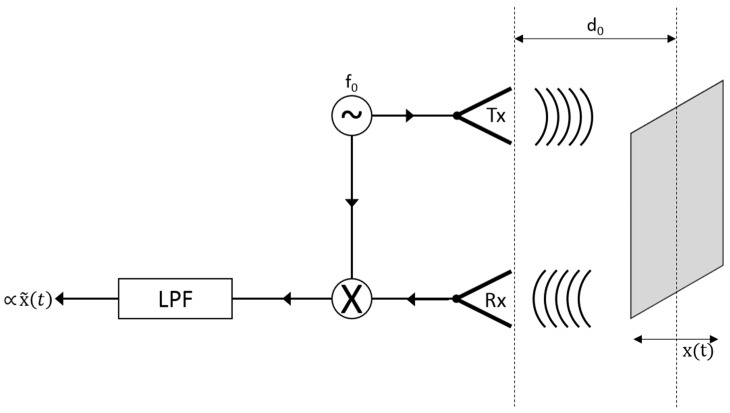
CW Doppler radar for vibration detection.

In the case of vibrations monitoring, the reference scenario can be simulated by assuming the presence of a target, intended to represent the object subject to vibrations, which moves with periodic motion around the position *d*_0_ (Figure 2). The backscattered signal received by the radar presents modulated amplitude, frequency and phase. Assuming very small target displacements due to vibrations, it is possible to approximate the received signal as described by Equation (7), where *x*(*t*) represents the periodic motion of the target:

(7) $$r_{x}\left(t\right)\approx A_{r}\sin\left(2\pi f_{0}t-\frac{4\pi d_{0}}{\lambda }-\frac{4\pi x\left(t\right)}{\lambda }\right).$$

This received signal includes a phase modulated by the periodic motion of the target. The mixing operation followed by the low-pass filter, acts, in this case, as a phase demodulator; therefore, the resulting signal is approximately proportional to the periodic displacement *x*(*t*) due to the vibration activity. Through the use of FFT, it is then possible to evaluate the detected vibration rate.

To demonstrate the above point, let us write Equation (7) in a more compact form as:

(8) $$r_{x}\left(t\right)\approx A_{r}\sin\left(2\pi f_{0}t-\frac{4\pi \tilde{x}\left(t\right)}{\lambda }\right),$$

where

(9) $$\tilde{x}\left(t\right)=d_{0}+x\left(t\right).$$

After applying the mixing and filtering operations described in Figure 2, the following expression is obtained:

(10) $$x_{LPF}\left(t\right)=-A_{r}\sin\left(\theta +\frac{4\pi x\left(t\right)}{\lambda }\right),$$

where

(11) $$\theta =\frac{4\pi d_{0}}{\lambda }.$$

As extensively treated in literature [23,24], various factors affect the value of *θ*, such as the phase shift at the reflection surface and any distance between the mixer and the antenna. In particular, we can distinguish two different conditions, namely the optimum point and the null point, the first one giving the optimum sensitivity for the detection of target oscillation. In the present formulation, due to the choice of a sine function in Equation (7), the optimum point happens when the angle *θ* is an integer multiple of *π*. In this case, if assuming small-angle approximation (motion very small as compared to the wavelength) from Equation (10), we obtain a signal directly proportional to the displacement, namely:

(12) $$x_{LPF}\left(t\right)=A_{r}\frac{4\pi x\left(t\right)}{\lambda }.$$

However, if the angle *θ* is multiple of *π*/2, the following expression is obtained:

(13) $$x_{LPF}\left(t\right)=-A_{r}\left\{1-{\left[\frac{4\pi x\left(t\right)}{\lambda }\right]}^{2}\right\}.$$

**Figure 3:**
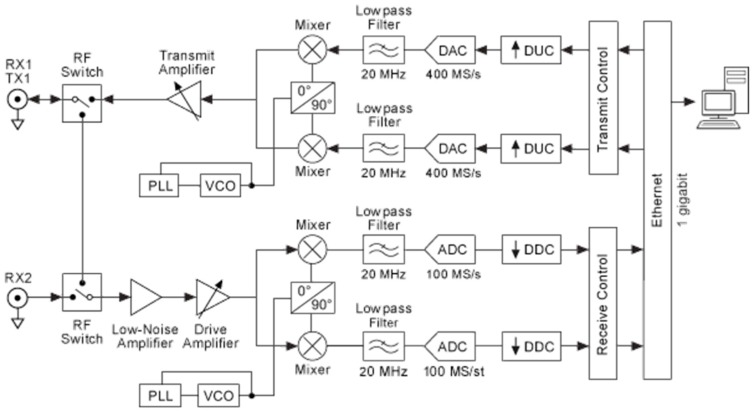
Block diagram of SDR platform. RX, Receiver; TX, Transmitter; VCO, Voltage-controlled oscillator; DAC, Digital-to-analog converter; DUC, Digital up converter; ADC, Analog-to-digital converter; DDC, Digital down converter.

In this case, the signal is no longer linearly proportional to the time-varying displacement, and the sensitivity is decreased [23], thus having the so-called null point. To avoid this undesired condition, the adoption of a quadrature receiver is suggested in literature [23]. This solution can be easily applied in our case, due to the specific architecture of an adopted SRD platform, which is based on an In-phase and Quadrature (I/Q) scheme (Figure 3). Furthermore, the presence of two oscillators including a Phase-Locked Loop (PLL) leads to neglecting the residual phase noise, as discussed in [23,24]. Anyway, the null detection point problem, which is of relevant importance for practical applications, will be accurately analyzed and faced in future studies.

Useful relationships can be highlighted between the radar parameters and its characteristics in terms of unambiguous capacity and resolution. In particular, the unambiguous capacity is determined from the low-pass cutoff frequency and the antenna reception bandwidth (Equation (14)), while from Equation (15), it is possible to determine the resolution (Equation (16)), where *T*_0_ stands for the duration of the acquisition. Therefore, the duration of the transmitted signal and the interval of reception:

(14) $$f_{max}=f_{T}=\frac{B}{2},$$

(15) $$N=T_{0}f_{s},$$

(16) $$\Delta f=\frac{f_{s}}{N}=\frac{f_{s}}{T_{0}f_{s}}=\frac{1}{T_{0}}.$$

## 4. Numerical Simulations

In order to demonstrate the validity of the proposed solution, an SDRadar platform is implemented in LabView software (v14.0, National Instruments, Austin, TX, USA) to simulate the operation and the processing flow performed by a CW Doppler radar, in order to demonstrate the system ability to make correct identifications of the oscillation frequency related to the vibrating target. For this purpose, the numerical elaborations are performed considering different target oscillation frequencies, with the target placed at different distances from the transmitting and the receiving antennas. The periodic displacement of the oscillating target is described by Equation (17), where *d*_0_ is the nominal distance at which the target is placed, *A* gives the amplitude of the displacement, obtained from relation of Equation (12), and *f_osc_* is the oscillation frequency:

(17) $$\tilde{x}\left(t\right)=d_{0}+A\sin\left(2\pi f_{osc}t\right).$$

For all simulations, the values of adopted input parameters *f*_0_ and B for the SDRadar platform are reported in Table 1. In particular, from the fixed bandwidth B, the unambiguous capacity *f_max_* is obtained by applying Equation (14).

**Figure 4:**
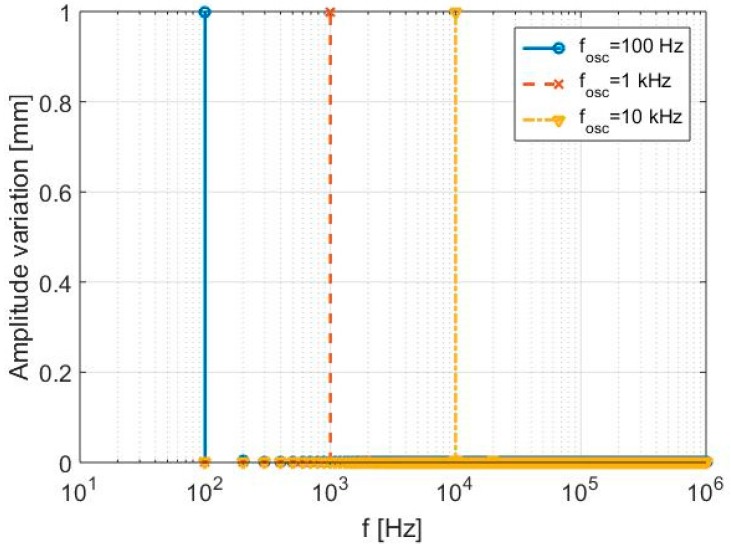
First simulation—Detected displacement spectra.

The first elaborations set is performed by assuming the target in a fixed position, equal to 10 cm, with fixed oscillation amplitude A and variable oscillation frequency *f_osc_* in a wide band, ranging from 100 Hz to 10 kHz. A fixed acquisition time *T*_0_ is imposed, thus giving a constant radar resolution ∆*f*. The adopted parameters and the achieved results are summarized in Table 2. In particular, the operations of mixing and filtering described in Figure 2 are applied to the received signal, assuming the form of Equation (7), to derive the displacement *x*(*t*), and, finally, the FFT processing is performed to retrieve the values of oscillation frequency *f_osc_* as reported in the sixth column of Table 2. These are visible in the amplitude spectra of Figure 4, which properly highlight how the proposed system is able to distinguish vibrations in a wide frequency range, with a very small relative error (0.2%) in the amplitude detection.

**Figure 5:**
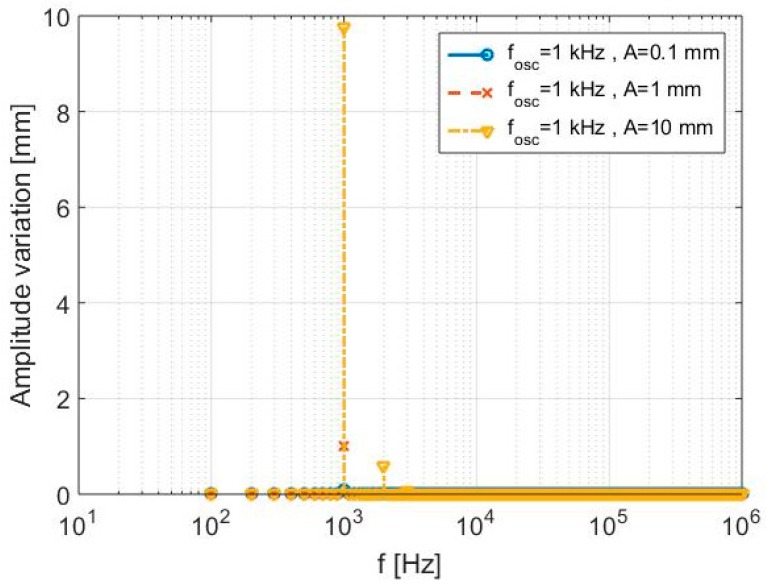
Second simulation—Detected displacement spectra.

A second numerical test is performed considering again the target in a fixed position, but with fixed oscillation frequency *f_osc_* = 1 kHz and variable oscillation amplitude A. The new elaboration results, summarized in Table 3 and shown in Figure 5, properly highlight the further sensitivity of the system to different amplitude oscillations. In this case, as evident from the spectra of Figure 5, the retrieved oscillation frequency *f_osc_* is exact and constant for all three tests, while a different spectrum amplitude is obtained, due to the different values of oscillation amplitude A. It can be also observed how the relative error in the amplitude reconstruction increases (but remain limited to 2.36%) as the imposed amplitude variation reaches the high value of 10 mm.

**Figure 6:**
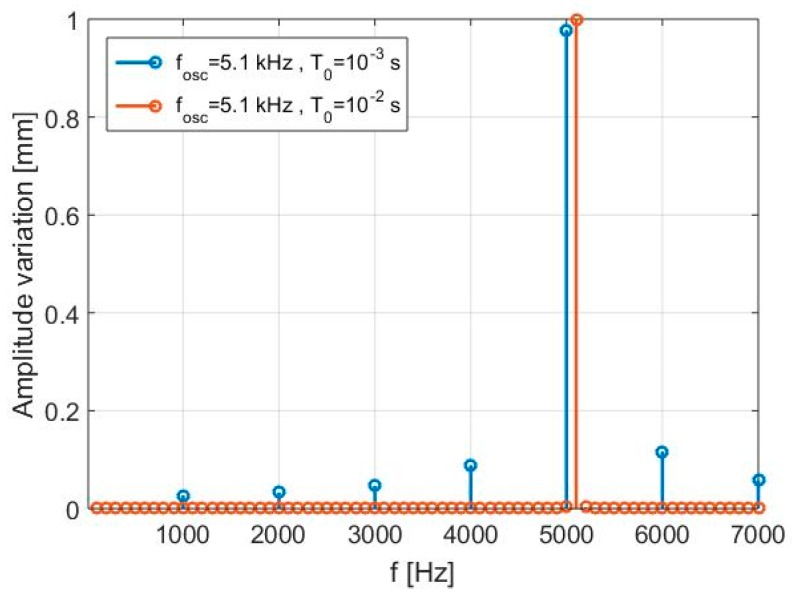
Third simulation—Detected displacement spectra.

A third numerical test is further performed to show the influence of the acquisition duration *T*_0_ on the resolution ∆*f*. The results, summarized in Table 4 and shown in Figure 6, indicate how the SDRadar platform is able to accurately detect the target oscillation frequency *f_osc_* = 5.1 kHz, once a proper time duration *T*_0_ = 10^−2^ s is fixed, and thus a proper resolution ∆*f*. As a matter of fact, the case illustrated in the first row of Table 4 prove that, when fixing a value *T*_0_ < 10^−2^ s, the oscillation frequency f_osc_ is not accurately retrieved, and we also have a relative error equal to 2.3% in the amplitude reconstruction.

## 5. Experimental Results

**Figure 7:**
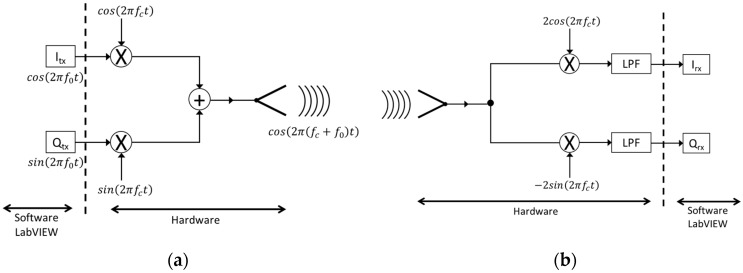
SDR NI USRP 2920 transceiver: (**a**) I/Q NI USRP 2920 Modulator; (**b**) I/Q NI USRP 2920 demodulator. LPF, Low-pass filter.

**Figure 8:**
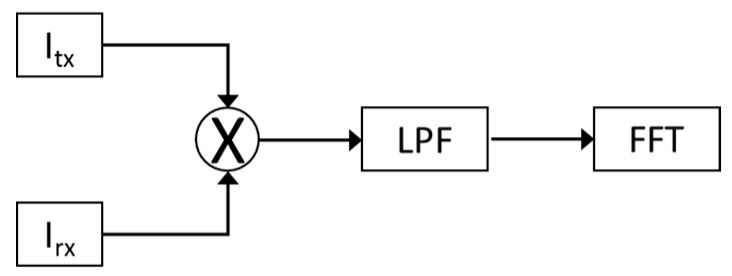
Processing algorithm performed on LabView software (v14.0, National Instruments, Austin, TX, USA).

As a final validation check of the proposed approach, a Doppler SDRadar platform is realized, and experimental tests are successfully assessed. The radar is essentially realized on the transceiver SDR NI USRP 2920 (National Instruments, Austin, TX, USA), directly interfaced to a PC for data acquisition and processing. A standard omni-directional dipole antenna in transmission, and a strong near-field Impinji A0303 (Impinj, Seattle, WA, USA) [25] antenna in reception, are adopted. The transceiver includes an IQ modulator and demodulator, on the TX and RX channels, respectively. The IQ components of the transmitted signal are generated through LabView code, as shown in Figure 7a, where *fc* gives the carrier frequency. At the receiver, the IQ components, extracted from the demodulator (Figure 7b), are acquired on the PC using LabView software and then processed according to the diagram in Figure 8.

In order to test the radar functionality, two distinct experimental validations are performed.

**Figure 9:**
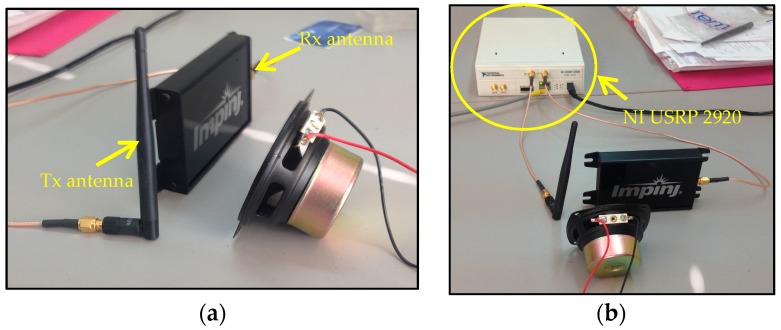
Experimental setup: (**a**) vibrating membrane in the presence of Tx and Rx antennas; (**b**) full SDRadar Doppler configuration.

**Figure 10:**
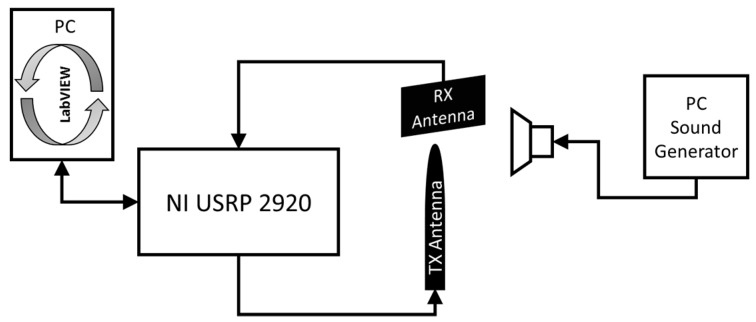
Experimental setup diagram.

As a first test, the displacement produced by the vibrating membrane of a standard speaker is considered. By connecting the speaker to the output of a PC sound card, through audio signal generation software, it is possible to produce a variable frequency membrane displacement. For a standard speaker, the membrane displacement *x*(*t*) is on the order of 0.1 mm. With reference to the scheme in Figure 2, the antennas and the target are placed at a mutual distance *d*_0_ equal to 5 cm (Figure 9a,b). The experimental setup diagram is shown in Figure 10.

**Figure 11:**
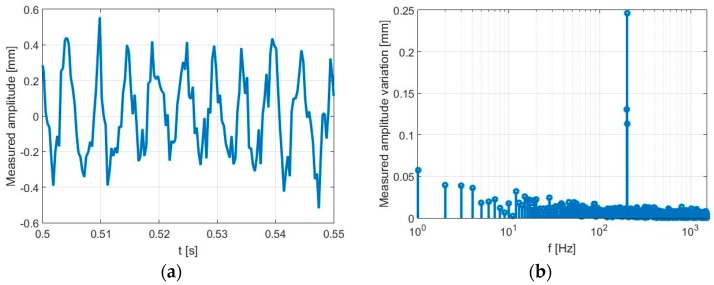
Captured scene in the presence of an oscillating target: (**a**) time domain; (**b**) frequency domain.

For the validation test, we set *f*_0_ = 20 kHz and *fc* = 1 GHz. Displacement data *x*(*t*) related to a captured scene (at a given time) are illustrated in the time domain (Figure 11a) as well as in the frequency domain (Figure 11b). In particular, in the second figure, it is easy to distinguish the maximum peak giving the oscillation frequency *f_osc_* of the membrane, equal to 200 Hz in the examined case.

**Figure 12:**
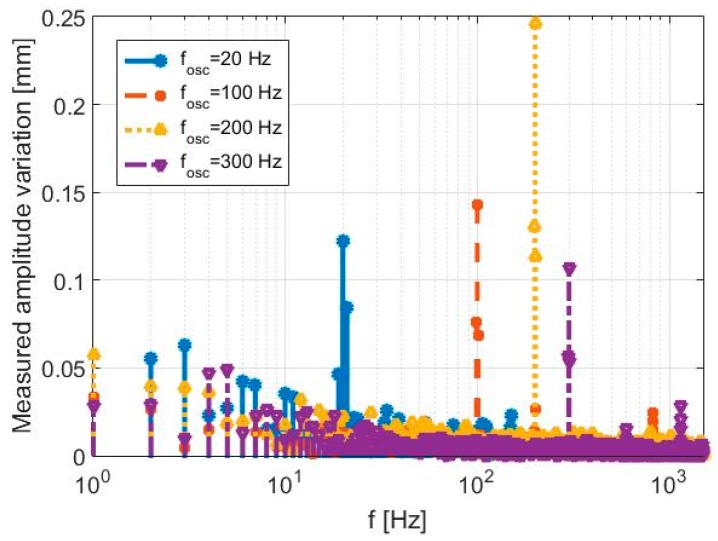
Parameters and results of the first experimental validation tests (first set).

The SDRadar Doppler platform is validated through various experimental tests, by producing vibrations with different oscillation frequencies. The related experimental results are summarized in Table 5 and compared in Figure 12. In particular, a relative error equal to zero is obtained in the reconstructed oscillation frequency, for all tests.

**Figure 13:**
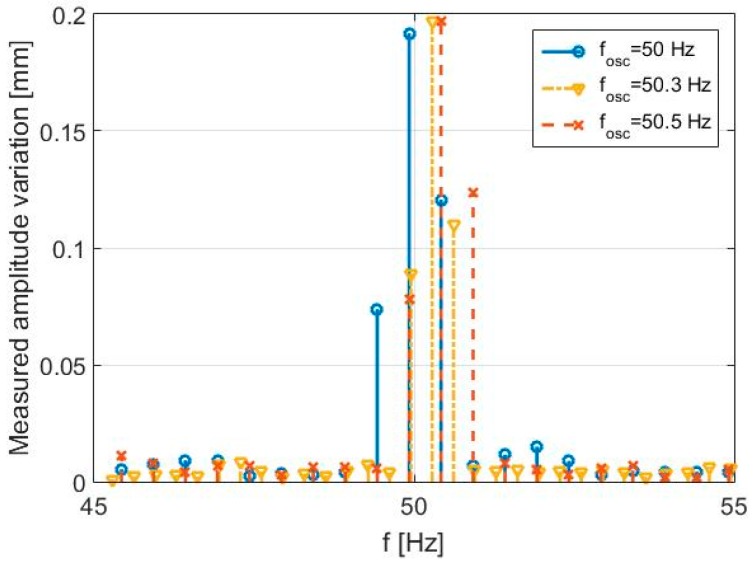
Parameters and results of the first experimental validation test (second test).

In order to verify the SDRadar ability in distinguishing targets with oscillation frequencies shifted by a single resolution step, the same experiment is performed with different acquisition intervals. The results are shown in Table 6 and reported in Figure 13. In particular, when properly increasing the acquisition time *T*_0_, a decrease of the relative error (from 0.16% down to 0.04%) is successfully observed.

**Figure 14:**
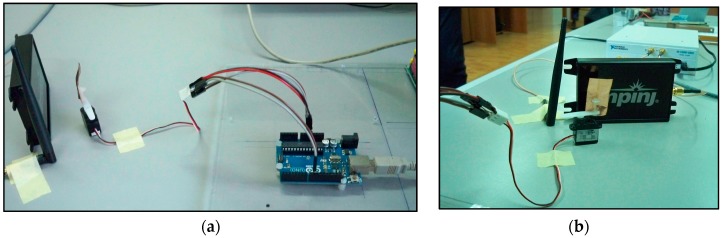
Setup of the second experimental test: (**a**) stepper micro motor connected to Arduino UNO (Arduino LLC, Ivrea, Italy); (**b**) stepper micro motor with a target in the presence of Tx and Rx antennas.

**Figure 15:**
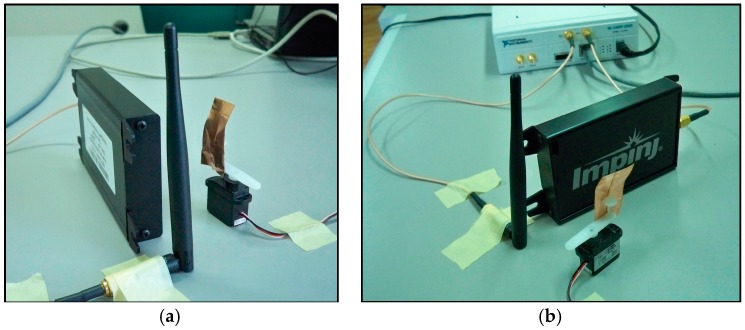
Setup of the second experimental test: (**a**) stepper micro motor with target in the presence of Tx and Rx antennas; (**b**) full SDRadar Doppler configuration on the second scenario.

**Figure 16:**
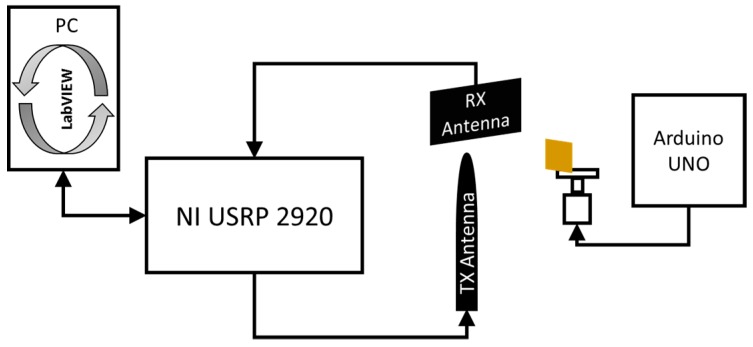
Setup diagram of the second experimental test.

The audio speaker as the one adopted in the first experimental test is able to produce vibrations limited to a minimum frequency of around 20 Hz. Thus, in order to verify the capabilities of the proposed SDRadar system at very low frequencies, a second experimental setup is considered. It is realized by applying a small metal target on the axis of a stepper micro motor controlled through an Arduino UNO (Arduino LLC, Ivrea, Italy) (Figure 14a,b). The antennas and the target are placed at the same distances as in the previous test (Figure 15a,b). The experimental setup diagram of this second test is shown in Figure 16.

**Figure 17:**
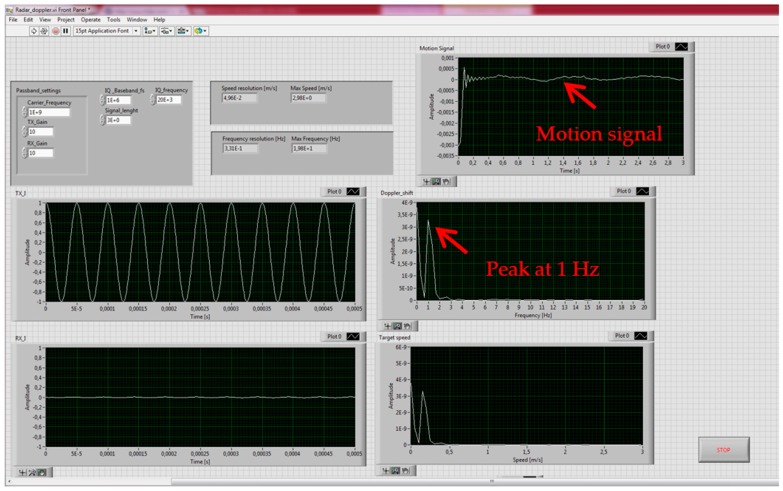
Screenshot of LabView software relative to the test case with oscillation frequency equal to 1 Hz.

A screenshot example of the SDRadar implementation (LabView software code), relative to the case of an oscillation frequency equal to 1 Hz, is illustrated in Figure 17.

**Figure 18:**
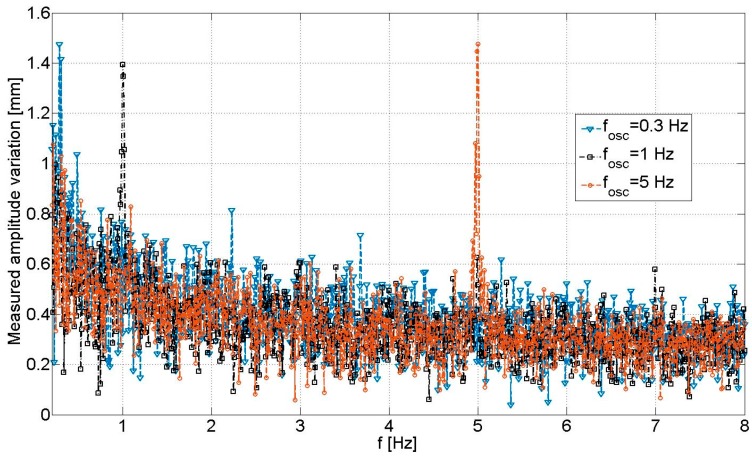
Parameters and results of the experimental validation tests on the second scenario.

A summary of all experimental results relative to the experimental setup of Figure 14, Figure 15 and Figure 16 is reported in Table 7, while the relative amplitude spectra are shown in Figure 18. The achieved results properly highlight the system capability to detect oscillations at frequencies below 10 Hz. In particular, a relative error equal to zero can be observed in the reconstruction of the oscillation frequency.

**Figure 19:**
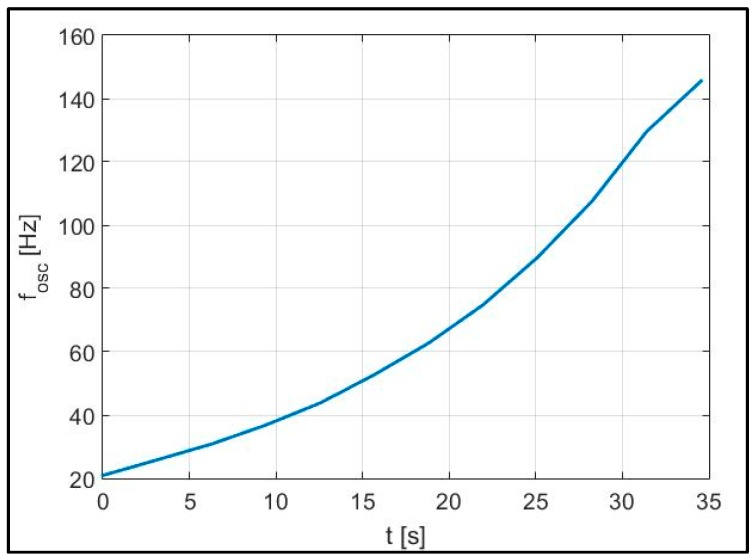
Monitoring of the target oscillation frequency within a time window of 35 s.

As a further test, the generated audio signal performing a frequency sweep is considered. By monitoring the maximum peak frequency within a time window equal to 35 s (Figure 19), an increase over time of the detected oscillation frequency *f_osc_* is obtained. 

Finally, as a quantitative analysis of the performances relative to the proposed SDRadar Doppler approach, the response time is evaluated for different values of the acquisition time *T*_0_. Data reported in Table 8, indicating the average value of the radar elaboration time and response time, are obtained from a set of 50 acquisitions for each value of assumed time *T*_0_.

## 6. Conclusions

In this work, an SDRadar Doppler platform has been implemented and proposed for the monitoring of target vibrations. In the first part of the paper, a detailed discussion of the theoretical background for a Continuous Wave Doppler Radar is provided, by deriving the expressions for the Doppler resolution and the unambiguous capacity of the proposed radar platform. Then, numerical simulations have been discussed to show the ability of the proposed approach to detect different kinds of vibrations, characterized by oscillations with different frequencies and amplitudes, related to targets placed at different distances. Afterwards, the realized SDRadar platform has been discussed, with a detailed description of the various components and operating schemes. Finally, experimental tests have been performed, by adopting a standard speaker to produce vibrations characterized by different oscillation frequencies in the range 20–300 Hz, thus proving the ability of the proposed SDRadar Doppler to detect very small vibrations, of the order of 0.1 mm.

These preliminary validations encourage the adoption of the realized SDRadar platform for contactless detection in various context fields, such as those including security and biomedical applications. As a matter of fact, the particular Software-Defined approach leads to detecting very low oscillation frequencies with high accuracy by simply changing via software the radar bandwidth B and the acquisition time *T*_0_. In the present work, a single tone movement is assumed, thus no spurious harmonic is expected; however, we deserve the extension of the proposed SDRadar Doppler platform to real physiological movements.

## Figures and Tables

**Table 1:** Fixed simulations parameters.

*f*_0_	B	*f_max_*
1 GHz	2 MHz	1 MHz

**Table 2:** First simulation—Parameters and results.

*A*	*d*_0_	*T*_0_	Δ*f*	*f_osc_*	*f_osc_* (Identified)	Relative Error (*f_osc_*)	Spectrum Amplitude	Relative Error (Amp)
1 mm	10 cm	10^−2^ s	100 Hz	100 Hz	100 Hz	0	0.998 mm	0.2%
1 mm	10 cm	10^−2^ s	100 Hz	1 kHz	1 kHz	0	0.998 mm	0.2%
1 mm	10 cm	10^−2^ s	100 Hz	10 kHz	10 kHz	0	0.998 mm	0.2%

**Table 3:** Second simulation—Parameters and results.

*A*	*d*_0_	*T*_0_	Δ*f*	*f_osc_*	*f_osc_* (Identified)	Relative Error (*f_osc_*)	Spectrum Amplitude	Relative Error (Amp)
0.1 mm	1 m	10^−2^ s	100 Hz	1 kHz	1 kHz	0	0.0998	0.2%
1 mm	1 m	10^−2^ s	100 Hz	1 kHz	1 kHz	0	0.998	0.2%
10 mm	1 m	10^−2^ s	100 Hz	1 kHz	1 kHz	0	9.764	2.36%

**Table 4:** Third simulation—Parameters and results.

*A*	*d*_0_	*T*_0_	Δ*f*	*f_osc_*	Relative Error (*f_osc_*)	*f_osc_* (Identified)	Spectrum Amplitude	Relative Error (Amp)
1 mm	10 cm	10^−3^ s	1 kHz	5.1 kHz	1.96%	5 kHz	0.977	2.3%
1 mm	10 cm	10^−2^ s	100 Hz	5.1 kHz	0	5.1 kHz	0.998	0.2%

**Table 5:** Parameters and results of the experimental validation tests (first set).

B	*f_max_*	*T*_0_	Δ*f*	*f_osc_*	*f_osc_* (Identified)	Measured Amplitude Variation
3 kHz	1.5 kHz	1 s	1 Hz	20 Hz	20 Hz	0.122 mm
3 kHz	1.5 kHz	1 s	1 Hz	100 Hz	100 Hz	0.143 mm
3 kHz	1.5 kHz	1 s	1 Hz	200 Hz	200 Hz	0.246 mm
3 kHz	1.5 kHz	1 s	1 Hz	300 Hz	300 Hz	0.107 mm

**Table 6:** Parameters and results of the first experimental validation tests (second set).

B	*f_max_*	*T*_0_	Δ*f*	*f_osc_*	*f_osc_* (Identified)	Measured Amplitude Variation
300 Hz	150 Hz	2 s	1/2 Hz	50 Hz	49.92 Hz	0.192 mm
300 Hz	150 Hz	2 s	1/2 Hz	50.5 Hz	50.42 Hz	0.197 mm
300 Hz	150 Hz	3 s	1/3 Hz	50.3 Hz	50.28 Hz	0.197 mm

**Table 7:** Parameters and results of the second experimental validation test.

B	*f_max_*	*T*_0_	Δ*f*	*f_osc_*	*f_osc_* (Identified)
3 kHz	1.5 kHz	100 s	0.01 Hz	0.3 Hz	0.3 Hz
3 kHz	1.5 kHz	100 s	0.01 Hz	1 Hz	1 Hz
3 kHz	1.5 kHz	100 s	0.01 Hz	5 Hz	5 Hz

**Table 8:** Radar elaboration time and response time.

*T*_0_ (s)	Radar Elaboration Time (s)	Response Time (s)
10^−3^	2.040	2.041
10^−2^	2.120	2.130
6 × 10^−1^	2.083	2.683
1	2.124	3.124
2	2.223	4.223
3	3.320	5.320
